# Study of the Parotid Gland

**Published:** 1860-07

**Authors:** Claude Bernard


### ARTICLE XX.

*Study of the Parotid Gland.* By M. Claude Bernard.

We have continued the series of experiments which we commenced on the motor nerve of the parotid gland. Our observations on it have been made without much difficulty on the present occasion—the natural consequence of our having previously discovered by dissection, during our first experiment, that it is a branch of the auriculo-temporal nerve. Now, you are perfectly aware that this branch of the fifth pair of nerves has innumerable anastomosing branches emanating from the filaments of the facial nerve. It is, therefore, evident that, far from contradicting the results of our experiments on the section of the facial nerve during our researches on it in the interior of the cranium, this last observation only goes to confirm them. The nerve which animates the parotid gland and presides over its secretion must consequently be one of the anastomosing branches furnished by the facial to the superficial auriculo-temporal nerve. On a level with the condyle of the jaw this latter furnishes a small branch, which pursues its course alongside the internal maxillary artery; but following a direction the inverse of that of the current of the blood: it then enters the parotid gland, where it ramifies freely. It is this bundle of nervous filaments which we must henceforth consider as the motor power of the parotid gland; and we must completely extirpate them in our dissection if we desire to arrest the secretion. The same results have been obtained in the course of our experiments on two other dogs.

"The discovery of the properties of this bundle of nerves presents under different points of view no small amount of interest. Call to mind the opinions which were formerly in vogue with reference to the parotid gland ; it was regarded as absolutely passive in the act of insalivation ; it was regarded by all physiologists as a kind of sponge, which the movements executed by the animal during mastication must have pressed in order to make the saliva flow. It is true that the gland was observed to become active during the trituration of alimentary substances, and no nervous filament was recognized the direct excitation of which was supposed to stimulate the secretion; the conclusion, therefore, seemed to be quite legitimate. To-day, you must observe, this opinion is no longer admissible ; for during our galvanization of the nerve, this filament, which is no longer serviceable (seeing that it is cut,) does not give rise to any pain ; no movement is produced ; the jaws are motionless : it is not, therefore, to mastication that we are to attribute the flow of saliva which follows the galvanization ; here there is an important fact which science has acquired.

"The parotid gland does not play an absolutely passive part in the insalivation of aliments.

"But this discovery may be turned to advantage in a more general point of view in our study of the circulation of the glands. The mechanism of the secretion was formerly explained by a hypothesis founded on purely mechanical grounds ; the pressure of the blood in the vessels which lead to the glands was supposed to determine through their sides a transudation which was believed to pour into the acini a special product for each of the secreting organs. Ludwig has clearly demonstrated the inadmissibility of this hypothesis; he proved that an amount of pressure much more considerable than that occasioned by the circulation of the blood could be applied to the glands without exciting in any way the activity of their secretion. He adapts a manometer to the excretory duct of the submaxillary gland, he then ties this canal so as to completely prevent the escape of the secreted products; he at length excites, by means of galvanism, the motor nerve of the gland, that is to say, the chorda tympani; operating in this manner, we obtain, by the accumulation of the saliva, a pressure which raises the mercury to thirty or even forty centimetres; this pressure is evidently directed in an inverse sense to that exercised by the blood; nevertheless, the secretion goes on uninterruptedly. The secretion of glands is, therefore, not due to a simple mechanical cause; and it is this which the rational study of the circulation in the minor vascular systems corresponding or communicating with the secreting bodies demonstrates.

"While the gland remains in a state of repose, its venous blood is black; but no sooner does it begin to act, than its blood becomes red, and like that of the arteries, and when the vein is cut, the blood is remarked to issue forth in jerks, as if an artery had been cut. It seems, therefore, at first sight, that the circulation of the gland is accelerated, and that the secretion is the result of this new modification; but these conditions alone do not suffice to determine the physiological phenomenon; it is still necessary that nervous action should come into play. When we excite the secretion by sensations produced by moist substances, it becomes very difficult to decide between the part played by the circulation in the interior of the gland and that which is produced in the surrounding parts; the animal executes movements of deglutition, moves the jaws and thus causes the blood to flow from all sides toward the vessels of the gland. Nothing of the like kind takes place when we apply direct excitation, by means of galvanism, to the motor nerve. The animal experiences not the slightest sensation ; he remains motionless, and the circulation proceeds with the most perfect regularity. It is then that the observer is placed in excellent conditions for studying it. We have already adopted a method of procedure, as regards this subject, in the case of the sub-maxillary gland, but it has been impossible for us, up to the present moment, to repeat the experiment on the parotid, and it is especially with a view to this object that we have undertaken a search for the motor filament of this latter gland ; for, if it were possible to compare, loupe and scalpel in hand, the act of secretion which takes place in two different glands, such, for example, as the parotid and the submaxillary, and to apprehend, at a glance, the difference and the analogy existing between them, an immense step would thus be made toward the discovery of the real essence of this important physiological function.

"The study of the properties of this nervous filament is fraught with interest in other respects : we know, for example, that by injecting into the torrent of the circulation certain substances, camphor for instance, the salivary secretion is powerfully excited ; this is a subject which it is our intention to investigate, and especially for the purpose of ascertaining the nervous action which may determine it.

"Formerly, during my investigation of the subject of diabetes produced by lesion of certain parts of the encephalon, I proved that a well-marked salivation could be produced in an animal simply by wounding certain definite points. The wound which determines these accidents in the economy must be made on the floor of the fourth ventricle. Now when the cutting instrument chances to deviate to the right or the left, instead of acting directly on the median line, there is observed to take place a diminution in the flow of saliva from the gland of the same side ; but when the wound is made directly over the median line the flow is the same on both sides.

"We have also remarked that the submaxillary gland furnishes more saliva than the parotid. To what is this difference to be attributed? The wound, as we stated, is made on the floor of the fourth ventricle, but the salivation takes place only when the point of the cutting instrument, being directed forward, wounds the parts adjoining the origin of the fifth pair of nerves. It would appear, therefore, that an injury bearing directly on the nervous centre, which presides over the salivary functions, immediately gives rise to the secretion dependent on it; in fact, we know that it is through the medium of the fifth pair of nerves that most of the reflex actions are produced, which exercise on these glands such a powerful influence; in this nerve may be said to terminate the greater part of the gustatory impressions which may be called the ordinary source of the salivary flow. We have frequently repeated an experiment which goes to confirm the truth of what we have just stated. Cut the lingual nerve, galvanize its peripheric extremity, no result follows; give vinegar to the animal, still no effect is produced; the nerve which should transmit the impression, being divided. But if you galvanize the other extremity of the nerve, thus acting directly on the nervous centre, you will produce a secretion similar to what is observed under ordinary circumstances; but if, instead of acting on the nervous centre by galvanization of the lingual nerve, you irritate directly the nervous centre itself, you will then produce a salivary diabetes, if I may be allowed to use such an expression.

"But, in addition to this, there exists certain poisons which act directly on the salivary glands; woorara, for example, which, as you know, kills very rapidly, by producing phenomena characterized by general paralysis, induces at the same time an abundant flow of saliva, which is to be explained, no doubt, by the complete relaxation which produces in the entire glandular tissue.

"It may be objected that the asphyxia which this poison causes of itself suffices to explain the phenomenon: in order completely to solve the question, I injected some woorara into one of the small arterial branches which anastomose with the artery of the gland, at the same time taking care to open all the veins. In this way I poisoned the acini of the gland without poisoning the animal; I thus obtained a flow of saliva which it was impossible could have been produced by any other cause than the woorara.

"Worrara acts, therefore, on the *salivary* glands in the same way as it does on the other glands of the economy. Now, we are aware that this toxic agent acts especially on the extremities of the motor nerves; but how does this action on the extremities of their nerves affect the glandular bodies themselves? This is a difficult question, which we shall endeavor to solve at some future time. It has been our object, on the present occasion, merely to show you that a nervous centre presides over the salivary function. Having shown you that a permanent salivation may be produced in animals by artificial means, it follows that there exists a special nerve whose duty it is to preside over the action of each of the glands; and the discovery which we have recently made completes the series of proofs on which this doctrine is founded."—*Med. Times and Gazette.*

